# Effect of beta blockers in acute and chronic coronary syndromes without reduced ejection fraction: a landmark analysis from the REBOOT trial

**DOI:** 10.1093/ehjcvp/pvag002

**Published:** 2026-01-22

**Authors:** Xavier Rossello, José A Barrabés, Massimo Piepoli, Alberto Dominguez-Rodriguez, Pedro L Sánchez, Manuel Anguita, Sergio Raposeiras-Roubín, Giulietta Grigis, Jaume Agüero, Ruth Owen, Stuart Pocock, Carlos Nicolás Pérez-García, Noemí Escalera, Andrea Kallmeyer, Alessandro Sionis, Lidia Staszewsky, Alfonso Torres, Rocio Barquero, Felipe Fernández-Vazquez, Francisco Marín, Alfredo Vetrano, Pablo Pastor, Valentín Fuster, Roberto Latini, Borja Ibanez

**Affiliations:** Clinical Research Department, Centro Nacional de Investigaciones Cardiovasculares Carlos III (CNIC), Madrid 28029, Spain; Centro de Investigación Biomédica en Red en Enfermedades Cardiovasculares -CIBERCV-, Madrid 28029, Spain; Cardiology Department, University Hospital Son Espases, Instituto de Investigación Sanitaria Islas Baleares and Universitat de les Illes Balears (UIB), Palma de Mallorca 07120, Spain; Centro de Investigación Biomédica en Red en Enfermedades Cardiovasculares -CIBERCV-, Madrid 28029, Spain; Department of Cardiology, Hospital Universitari Vall d´Hebron, Barcelona 08035, Spain; Cardiology Department, Ospedale Guglielmo da Saliceto, Piacenza 29100, Italy; Centro de Investigación Biomédica en Red en Enfermedades Cardiovasculares -CIBERCV-, Madrid 28029, Spain; Department of Internal Medicine, Universidad de La Laguna, Instituto de Investigación Sanitaria de Canarias, Tenerife 38320, Spain; Department of Cardiology, Hospital Universitario de Canarias, Instituto de Investigación Sanitaria de Canarias, Tenerife 38320, Spain; Centro de Investigación Biomédica en Red en Enfermedades Cardiovasculares -CIBERCV-, Madrid 28029, Spain; Department of Cardiology, Hospital Universitario de Salamanca, Biomedical Research Institute of Salamanca (IBSAL), Salamanca 37007, Spain; Centro de Investigación Biomédica en Red en Enfermedades Cardiovasculares -CIBERCV-, Madrid 28029, Spain; Departament of Cardiology, Hospital Universitario Reina Sofía, Instituto Maimónides de Investigación Biomédica de Córdoba (IMIBIC), University of Córdoba, Córdoba 14004, Spain; Clinical Research Department, Centro Nacional de Investigaciones Cardiovasculares Carlos III (CNIC), Madrid 28029, Spain; Cardiology Department, Hospital Universitario Alvaro Cunqueiro, Vigo 36312, Spain; Dipartimento Di Cardiologia, Ospedale Bolognini, Seriate 24068, Italy; Centro de Investigación Biomédica en Red en Enfermedades Cardiovasculares -CIBERCV-, Madrid 28029, Spain; Cardiology Department, Hospital Universtitari i Politecnic La Fe, Valencia, Spain; Clinical Research Department, Centro Nacional de Investigaciones Cardiovasculares Carlos III (CNIC), Madrid 28029, Spain; Department of Medical Statistics, London School of Hygiene and Tropical Medicine, WC1E 7HT London, UK; Clinical Research Department, Centro Nacional de Investigaciones Cardiovasculares Carlos III (CNIC), Madrid 28029, Spain; Department of Medical Statistics, London School of Hygiene and Tropical Medicine, WC1E 7HT London, UK; Clinical Research Department, Centro Nacional de Investigaciones Cardiovasculares Carlos III (CNIC), Madrid 28029, Spain; Clinical Research Department, Centro Nacional de Investigaciones Cardiovasculares Carlos III (CNIC), Madrid 28029, Spain; Centro de Investigación Biomédica en Red en Enfermedades Cardiovasculares -CIBERCV-, Madrid 28029, Spain; Cardiology Department, University Hospital Fundación Jiménez Díaz and Instituto de Investigación Sanitaria-Fundación Jiménez Díaz (IIS-FJD, UAM), Madrid 28040, Spain; Cardiology Department, Hospital de la Santa Creu i Sant Pau, Barcelona 08025, Spain; Department of Acute Brain and Cardiovascular Injury, Istituto di Ricerche Farmacologiche Mario Negri IRCCS, Milan 20156, Italy; Cardiology Department,Hospital Txagorritxu, Vitoria-Gasteiz 01009, Spain; Cardiology Department, Hospital Universitario Virgen de la Macarena, Sevilla 410042, Spain; Cardiology Department, Hospital Universiario de León, León 24008, Spain; Cardiology Department, Hospital Virgen de la Arrixaca, IMIB-Arrixaca and University of Murcia, Murcia 30120, Spain; Cardiology Department, Ospedale S. Anna e S. Sebastiano, Caserta 81100, Italy; Department of Cardiology, Arnau de Vilanova University Hospital, Lleida, Spain; Grup de Fisiologia i Patologia Cardíaca, Institut de Recerca Biomèdica de Lleida Fundació Dr. Pifarré, IRBLleida, Lleida 25198, Spain; Clinical Research Department, Centro Nacional de Investigaciones Cardiovasculares Carlos III (CNIC), Madrid 28029, Spain; Mount Sinai Fuster Heart Hospital, New York, 10029 NY, USA; Department of Acute Brain and Cardiovascular Injury, Istituto di Ricerche Farmacologiche Mario Negri IRCCS, Milan 20156, Italy; Clinical Research Department, Centro Nacional de Investigaciones Cardiovasculares Carlos III (CNIC), Madrid 28029, Spain; Centro de Investigación Biomédica en Red en Enfermedades Cardiovasculares -CIBERCV-, Madrid 28029, Spain; Cardiology Department, University Hospital Fundación Jiménez Díaz and Instituto de Investigación Sanitaria-Fundación Jiménez Díaz (IIS-FJD, UAM), Madrid 28040, Spain

**Keywords:** beta blockers, Acute coronary syndrome, Chronic coronary syndrome, Randomized controlled trial, Landmark analysis

## Abstract

**Aims:**

Current guidelines recommend beta-blocker therapy after myocardial infarction (MI) regardless of left ventricular ejection fraction (LVEF). However, recent trials question their benefit in patients with preserved LVEF. No study has yet compared beta-blocker effects during the acute coronary syndrome (ACS) phase (≤1 year post-MI) vs. the chronic coronary syndrome (CCS) phase (>1 year).

**Methods and results:**

In this pre-specified landmark analysis of the REBOOT trial, we evaluated the effect of beta-blocker therapy on outcomes in two post-MI phases: the ACS period (first year; cohort 1, *n* = 8438) and the CCS period (>1 year, event-free patients with follow-up; cohort 2, *n* = 7783). The primary endpoint was all-cause death, nonfatal reinfarction, or heart failure hospitalization; secondary endpoints included individual and additional cardiovascular events. Among 623 primary outcome events, 238 occurred in the first year (28.9/1000 patient-years) and 385 thereafter (19.3/1000 patient-years). Secondary prevention use was generally high, but patients with early events had lower prescription rates than those with late events or no events. Beta-blockers were not associated with lower risk of the primary or component outcomes in either phase. A nonsignificant trend towards benefit of beta-blockers appeared during the first year in patients with mildly reduced LVEF (41–49%), whereas in the CCS phase, higher beta-blocker doses were associated with worse outcomes.

**Conclusion:**

In invasively treated MI patients with LVEF >40%, beta-blockers did not reduce adverse outcomes in either the ACS or CCS phases. These findings challenge their routine use in this population and support reconsidering current guidelines. Long-term beta-blocker users after MI may be candidates for deprescription.

## Introduction

Beta-blockers have been routinely used as part of the secondary prevention strategy following acute myocardial infarction (MI) since the 1980s.^1^ Early randomized controlled trials (RCT) reported a remarkable risk reduction in mortality at a time when reperfusion, complete revascularization, high-sensitivity troponin assays, and use of potent dual antiplatelet therapy (DAPT) and statins were not available.^2^ Moreover, those old RCTs included a mix of patients with uncomplicated MI with preserved left ventricular ejection fraction (LVEF) and MI patients with poor LVEF and/or heart failure (HF). Although the average follow-up of these old trials rarely went beyond 1 year,^2^ beta-blocker prescription was adopted as a life-long intervention.

Recently, both the European and American guidelines for the treatment of patients with acute coronary syndrome (ACS) have been updated. The 2023 European Society of Cardiology (ESC) ACS guidelines recommend a routine beta-blocker use for all patients regardless of LVEF (class IIa), although it acknowledged that the duration of beta-blocker therapy was a controversial topic.^2^ The more recent 2025 ACC/AHA/ACEP/NAEMSP/SCAI ACS guidelines recommend early (<24 h) initiation of beta-blockers in all ACS patients to reduce risk of reinfarction and ventricular arrhythmias (class I),^3^ with a reassessment of this therapy after 1 year (class 2b).^4^

Four contemporary pragmatic trials have tested the benefit of beta-blockers in post-MI patients treated according to current standards. Three of them showed no benefit of beta-blockers^5-7^ and a fourth one suggested a reduction in reinfarction rate associated with beta-blockers.^8^ At the time of ESC 2023 ACS guidelines release,^9^ no large contemporaneous trial in the topic was available, while at the time of American ACS guidelines release,^3^ only one large trial was available.^6^ Given this was a single trial, the ACC/AHA guideline decided to wait for additional evidence before changing a long-lasting recommendation. The REDUCE-AMI (Randomized Evaluation of Decreased Usage of Beta-Blockers after Acute Myocardial Infarction) trial in 5020 patients with MI (both STEMI and NSTEMI) and LVEF > 50% showed that beta-blocker therapy was not associated with a reduction of the risk of all-cause death or reinfarction over a 3.5 years median follow-up.^6^ The more recent and larger REBOOT (Effect of beta-blockers in TREatment with Beta-blockers after myOcardial infarction withOut reduced ejection fraction) trial in 8505 patients with MI (both STEMI and NSTEMI) and LVEF > 40% has shown that beta-blocker therapy is not associated with a reduction in the incidence of death, reinfarction or HF admission over a 3.7 years median follow-up.^5^ Paradoxically, the also recent ABYSS (Assessment of Beta-Blocker Interruption 1 Year after an Uncomplicated Myocardial Infarction on Safety and Symptomatic Cardiac Events Requiring Hospitalization) trial that randomized 3698 patients who were on beta-blockers for a median of 3 years after MI to stop or continue them concluded that beta-blocker withdrawal was not non-inferior to their maintenance.^10^ Despite a heterogeneity in findings from observational studies,^11–13^ some of them suggest that beta-blockers confer no benefit beyond 1 year after MI.^10,11^

To provide evidence from a modern large prospective RCT on the effect of beta-blockers in the first year after MI (ACS period) or beyond 1 year [chronic coronary syndrome (CCS) period], we analysed data from the recent REBOOT trial.

## Methods

### Study design

REBOOT was a pragmatic controlled prospective randomized open blinded-end point (PROBE) trial conducted at 109 centres across Spain and Italy, testing the benefits of beta-blockers in patients discharged from an uncomplicated MI and without reduced ejection fraction.^14^ Treatment allocation was not masked, but clinical outcomes were centrally adjudicated by a committee blinded to treatment allocation.^14^ Primary results of the REBOOT trial are published elsewhere.^5^ This investigator-initiated clinical trial was sponsored by the Centro Nacional de Investigaciones Cardiovasculares Carlos III (CNIC) in Madrid, Spain. The trial protocol was registered at clinicaltrials.gov (NCT03596385) and the European Clinical Trials Database (EUDRACT 2017-002485-40). Patient data were recorded in accordance with national personal data laws. The protocol was approved by the relevant ethics committees in Spain (EC 79-17/FJD) and Italy (2085, Prot.9144/2018; I.5/109) and adhered to the principles of the Declaration of Helsinki and the International Conference on Harmonization Good Clinical Practice guidelines. All participants provided written informed consent.

### Study population and intervention

Patients with MI [with or without ST-segment elevation (STEMI/NSTEMI)] were eligible if they underwent invasive management (i.e. coronary angiography) during the index episode and had a LVEF > 40% at discharge. The main exclusion criteria were a history of HF (including a killip-class ≥ II during index admission), a contraindication to or an indication for beta-blocker therapy unrelated to MI as determined by the treating physician. Further information on the study design and inclusion and exclusion criteria can be found elsewhere.^5,14^

Randomization was performed 1:1 (beta-blockers vs. no beta-blockers) at the time of hospital discharge or within the subsequent 14 days. For the present analysis, we defined two cohorts based on the 1-year landmark. Cohort 1 comprised patients with follow-up between randomization and 1 year, while cohort 2 comprised the period from 1 year onwards in patients who were event-free at the 12 months landmark. Following a pragmatic (close to real-life) design, in the intervention group, the type and dose of beta-blocker was at the discretion of the managing physician.

### Study outcomes

The primary outcome of the REBOOT trial was a composite of all-cause death, reinfarction or HF admission.^14^ Secondary outcomes included the individual components of the primary endpoint, cardiac death, sustained ventricular tachycardia, ventricular fibrillation, and resuscitated cardiac arrest.

Study endpoints for the present landmark analysis were the same primary and secondary outcomes of the main trial.

### Data analysis

Patient characteristics, in-hospital management, and medications at index date are reported as mean (standard deviation) or median (interquartile range) for continuous variables and as frequencies with percentages for categorical variables. Comparisons were made between those having a primary outcome within and beyond 1-year post-MI using either chi-square tests, *t* tests, or Wilcoxon rank sum tests, as appropriate.

The primary and secondary outcomes were analysed according to the intention-to-treat principle using proportional hazard models to generate unadjusted hazard ratios (HR) and 95% confidence intervals, also with a log-rank test of significance for the primary composite outcome.^15^ Follow-up for cohort 1 was from randomization to 1 year and from 1 year to end of trial follow-up in cohort 2. Patients who experienced the specific outcome during the first year were censored and therefore excluded from the analysis beyond 1 year (Cohort 2). A sensitivity per-protocol analysis was performed, with patient follow-up censored at the point of known crossover (i.e. beta-blocker patient stopped taking it or non-beta-blocker patient started taking it). Further information of crossovers is provided in the main trial publication.^5^ Additional analyses in pre-specified subgroups were performed for the primary endpoint stratified by LVEF (41–49% vs. ≥ 50%), and by beta-blocker dose (no beta-blocker *vs.* beta-blocker dose ≤ median vs. >median). Results for secondary end points are presented without formal adjustment for multiplicity.^16^ Complete case analyses were performed. A *P* value of less than 0.05 was considered statistically signiﬁcant. All analyses were performed using Stata (version 18.5).

## Results

### Study populations and distribution of primary events over time

**Figure 1:**
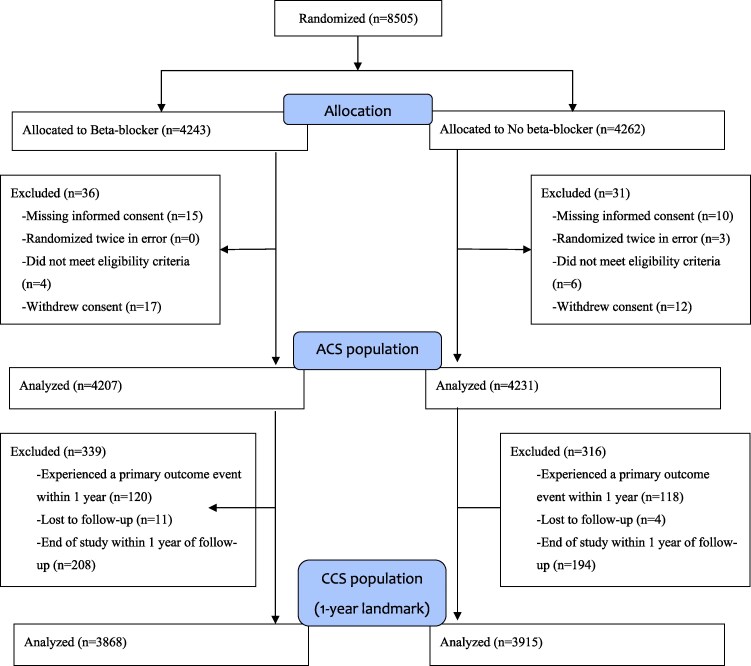
CONSORT diagram.

As shown in the flowchart (*Figure 1*), after exclusions, 8438 of the 8505 randomized patients were included in the intention-to-treat analysis, comprising Cohort 1 (ACS population). Baseline data by treatment allocation was published elsewhere.^5^ A total of 7783 patients who were free of the primary outcome event at 1 year and had follow-up beyond this time point comprised Cohort 2 (CCS population). The remaining 655 patients at risk during the first year did not enter the second observational period, primarily because they experienced a primary outcome event (*n* = 238) or lacked follow-up beyond 1 year (*n* = 417).

After a median follow-up of 3.7 years, a total of 623 primary endpoints occurred. Of these, 238 occurred within the first year (28.9/1000 patient-years), and 385 occurred beyond the first year (19.3/1000 patient-years): 120 during the second year (1–2 years), 101 during the third year (2–3 years), and 164 thereafter. Baseline characteristics according to the presence of the primary outcome event within and beyond the first year are presented in *Table 1*.

**Table 1:** Baseline characteristics in patients without event, and with event within and beyond the first year

	No event (*n* = 7815)	Event ≤1 year (*n* = 238)	Event > 1 year (*n* = 385)	*P* * (event ≤1 vs. >1y)
**Demographics**				
Age (years)	60.9 (10.9)	67.0 (12.7)	67.2 (12.4)	0.88
Country, *n* (%)				0.60
Spain	6070 (77.7)	178 (74.8)	295 (76.6)	
Italy	1745 (22.3)	60 (25.2)	90 (23.4)	
**Medical history**	***n* (%)**	***n* (%)**	***n* (%)**	
Hypertension	3956 (50.8)	155 (65.1)	256 (66.7)	0.69
Diabetes mellitus	1578 (20.3)	81 (34.0)	135 (35.4)	0.72
Dyslipidemia	3989 (51.2)	129 (54.4)	206 (53.8)	0.88
Smoking status				0.41
Non-smoker	2466 (32.4)	82 (36.1)	120 (32.4)	
Smoker	3447 (45.3)	77 (33.9)	151 (40.8)	
Ex-smoker <1yr	467 (6.1)	20 (8.8)	28 (7.6)	
Ex-smoker >1yr	1233 (16.2)	48 (21.1)	71 (19.2)	
Prior MI	664 (8.5)	55 (23.1)	83 (21.6)	0.66
Prior stroke	114 (1.5)	9 (3.8)	30 (7.8)	0.046
Atrial fibrillation prior to admission	149 (1.9)	21 (8.8)	23 (6.0)	0.18
Peripheral arterial disease	189 (2.4)	21 (8.8)	29 (7.6)	0.57
History of COPD	230 (2.9)	16 (6.7)	32 (8.3)	0.47
Treatment with beta-blockers before index admission	880 (11.3)	50 (21.1)	89 (23.2)	0.53
**Index admission**	***n* (%)**	***n* (%)**	***n* (%)**	
MI type, *n* (%)				0.44
STEMI	4028 (51.5)	107 (45.0)	161 (41.8)	
NSTEMI	3787 (48.5)	131 (55.0)	224 (58.2)	
Infarct related artery, *n* (%)				0.74
None	197 (2.5)	7 (3.0)	9 (2.3)	
Left anterior descending	2107 (27.0)	64 (27.2)	103 (26.8)	
Left circumflex system	971 (12.5)	23 (9.8)	32 (8.3)	
Right coronary artery	2050 (26.3)	48 (20.4)	101 (26.3)	
Secondary	429 (5.5)	13 (5.5)	18 (4.7)	
Left main	53 (0.7)	2 (0.9)	5 (1.3)	
Multivessel	1983 (25.5)	78 (33.2)	116 (30.2)	
Type of revascularization, *n* (%)				0.94
None	356 (4.6)	15 (6.4)	26 (6.8)	
PCI—stent	7151 (92.3)	211 (89.4)	342 (90.0)	
Complete revascularization, *n* (%)	6481 (88.7)	185 (84.1)	282 (81.7)	0.47
Included in cardiac rehabilitation programme, *n* (%)	2296 (33.3)	41 (20.1)	99 (29.5)	0.016
**Echo and laboratory tests**	**mean (SD)**	**mean (SD)**	**mean (SD)**	
LVEF (%), mean (SD)				
Creatinine prior to discharge (mg/dL), median (IQR)	0.9 (0.8–1.0)	0.9 (0.8–1.2)	0.9 (0.8–1.1)	0.45
eGFR (mL/min/1.73 m^2^), mean (SD)	90.5 (17.8)	80.2 (25.5)	82.8 (22.3)	0.18
Haemoglobin prior to discharge (g/dL), mean (SD)	14.3 (1.6)	13.4 (2.0)	13.7 (2.0)	0.037
**Discharge medication**	***n* (%)**	***n* (%)**	***n* (%)**	
Aspirin	7706 (98.7)	224 (94.9)	371 (96.4)	0.38
P2Y12 inhibitors	7653 (98.0)	223 (94.5)	373 (96.9)	0.14
ACE-i/ARB II	5845 (75.0)	171 (72.8)	293 (76.3)	0.32
Statins	7692 (98.6)	223 (94.5)	376 (97.7)	0.038
Aldosterone receptor antagonist	153 (2.0)	9 (3.8)	15 (3.9)	0.98
Oral anticoagulants	266 (3.4)	36 (15.3)	32 (8.3)	0.0068
Diuretics	677 (8.7)	45 (19.1)	54 (14.1)	0.094
Beta-blockers	3815 (48.8)	117 (49.2)	195 (50.6)	0.72
Type of beta-blocker, *n* (%)				0.47
Atenolol	24 (0.6)	0 (0.0)	2 (1.0)	
Bisoprolol	3288 (86.1)	98 (83.8)	163 (83.6)	
Carvedilol	113 (3.0)	5 (4.3)	10 (5.1)	
Metoprolol	285 (7.5)	11 (9.4)	13 (6.7)	
Nebivolol	105 (2.7)	2 (1.7)	7 (3.6)	
Other	4 (0.1)	1 (0.9)	0 (0.0)	
Beta-blocker dose				0.032
≤Median dosage	3294 (86.6)	100 (85.5)	146 (75.3)	
>Median dosage	508 (13.4)	17 (14.5)	48 (24.7)	

ACE-i, angiotensin-converting enzyme inhibitors; ARB, angiotensin receptor blockers.

^*^ *P* values are comparing patients with an event≤1 year to those with an event > 1 year.

### Clinical endpoints

**Figure 2:**
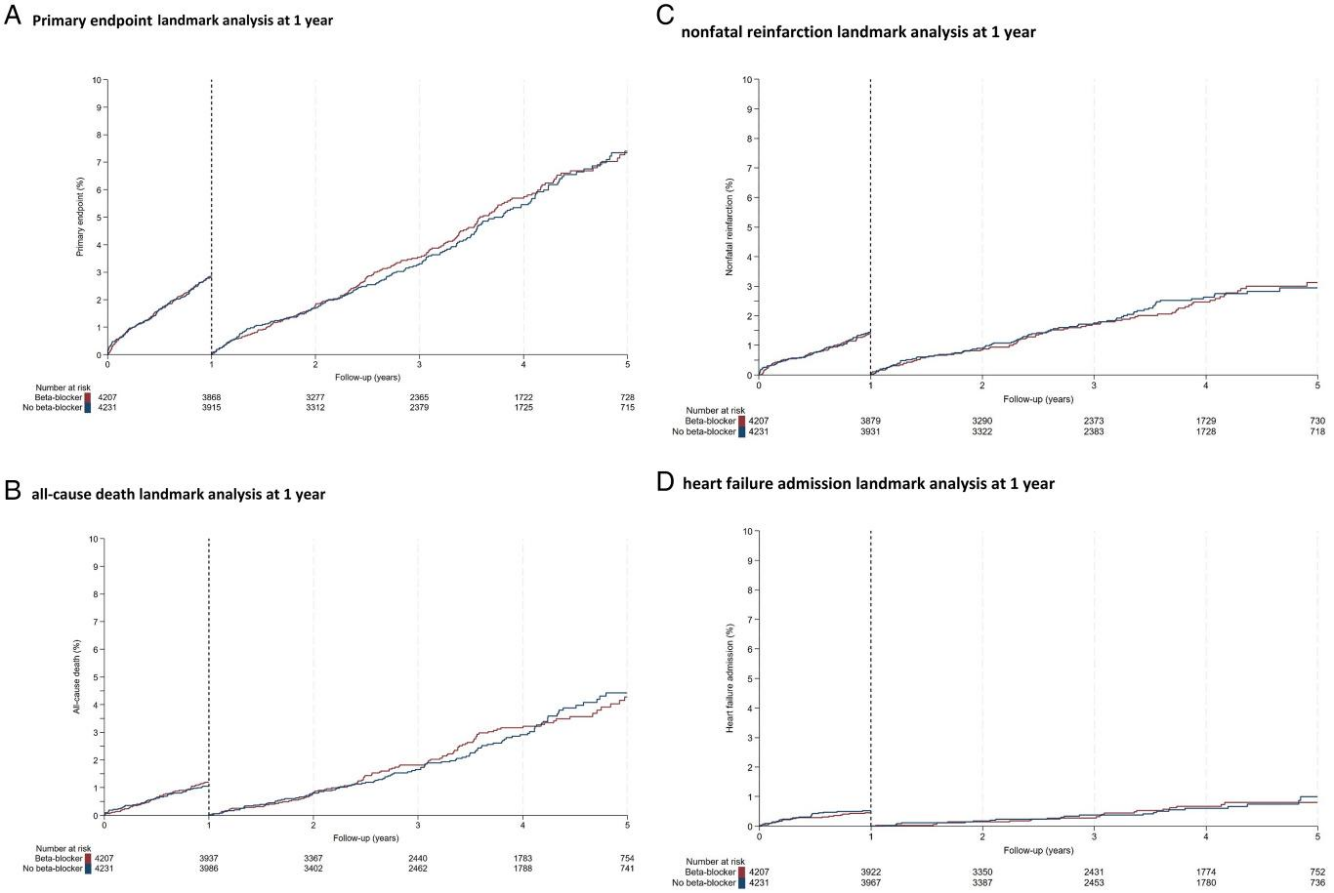
Kaplan–Meier plot for primary endpoint landmark analysis at 1 year and its individual components. *(A*) Primary endpoint. *(B*) all-cause death landmark analysis at 1 year. *(C*) nonfatal reinfarction landmark analysis at 1 year. *(D*) heart failure admission landmark analysis at 1 year.

Within the first year, the primary composite outcome of all-cause death, non-fatal reinfarction, or HF admission occurred in 120 participants (29.3/1000 patient-years) in the beta-blocker group and in 118 patients (28.5/1000 patient-years) in the no beta-blocker group (HR 1.02; 95% CI, 0.79 to 1.32; *P* = 0.85). Beyond the first year post-MI, the primary composite endpoint happened in 196 participants (19.8/1000 patient-years) in the beta-blocker group and in 189 patients (18.9/1000 patient-years) in the no beta-blocker group (HR 1.05; 95% CI, 0.86 to 1.28; *P* = 0.64). Survival curves for the primary outcome and its individual components according to the landmark are shown in *Figure 2*.

A similar lack of association between beta-blocker use, and clinical benefit was observed for individual outcomes all-cause death, cardiac death, non-cardiac death, nonfatal reinfarction, HF admission, admission for stroke, and unplanned revascularization (*Table 2*). There were no statistical differences in endpoints with small number of events (sustained ventricular tachycardia, ventricular fibrillation, resuscitated cardiac arrest, and admission for symptomatic advanced AV block), which are reported in Supplementary material online, *Table S1*.

**Table 2:** Landmark analysis at 1 year of the primary and key secondary endpoints

		*n* (rate per 1000 person-years)		Rate difference (95% CI)	HR (95% CI)	*P*
		Beta-blocker	No beta-blocker			
**Primary endpoint**						
All-cause death, nonfatal reinfarction and heart failure admission	Within 1 year	120 (29.3)	118 (28.5)	0.71 (−6.64, 8.05)	1.02 (0.79, 1.32)	0.85
	Beyond 1 year	196 (19.8)	189 (18.9)	0.89 (−2.97, 4.75)	1.05 (0.86, 1.28)	0.64
**Individual components**						
All-cause death	Within 1 year	50 (12.1)	44 (10.5)	1.53 (−3.04, 6.10)	1.14 (0.76, 1.72)	0.51
	Beyond 1 year	111 (10.9)	109 (10.6)	0.31 (−2.52, 3.14)	1.03 (0.79, 1.34)	0.82
Cardiac death	Within 1 year	22 (5.3)	20 (4.8)	0.52 (−2.53, 3.58)	1.11 (0.60, 2.03)	0.74
	Beyond 1 year	43 (4.2)	37 (3.6)	0.62 (−1.08, 2.33)	1.17 (0.76, 1.82)	0.47
Non-cardiac death	Within 1 year	28 (6.8)	24 (5.7)	1.01 (−2.39, 4.41)	1.18 (0.68, 2.03)	0.56
	Beyond 1 year	68 (6.7)	72 (7.0)	−0.32 (−2.57, 1.94)	0.96 (0.69, 1.33)	0.80
Nonfatal reinfarction	Within 1 year	59 (14.4)	60 (14.5)	−0.12 (−5.30, 5.06)	0.99 (0.69, 1.42)	0.96
	Beyond 1 year	84 (8.4)	83 (8.3)	0.17 (−2.36, 2.70)	1.02 (0.75, 1.38)	0.89
Heart failure admission	Within 1 year	19 (4.6)	22 (5.3)	−0.69 (−3.71, 2.34)	0.87 (0.47, 1.61)	0.65
	Beyond 1 year	20 (2.0)	22 (2.1)	−0.17 (−1.42, 1.07)	0.92 (0.50, 1.68)	0.78
**Other endpoints**						
Admission for stroke	Within 1 year	10 (2.4)	7 (1.7)	0.74 (−1.21, 2.69)	1.44 (0.55, 3.78)	0.46
	Beyond 1 year	27 (2.7)	18 (1.7)	0.91 (−0.38, 2.20)	1.52 (0.84, 2.76)	0.16
Unplanned revascularization	Within 1 year	71 (17.3)	74 (17.9)	−0.58 (−6.30, 5.15)	0.97 (0.70, 1.34)	0.84
	Beyond 1 year	99 (10.0)	97 (9.7)	0.27 (−2.49, 3.03)	1.03 (0.78, 1.36)	0.85

Hazard ratios were estimated using Cox proportional hazards models and estimate the effect of taking beta-blocker vs. no beta-blocker. *P* values were calculated using logrank tests. Endpoints with small number of events (sustained ventricular tachycardia, ventricular fibrillation, resuscitated cardiac arrest, and admission for symptomatic advanced AV block) are reported in Supplementary material online, *Table S1*.

The number of patients for the analysis of each outcome in the CCS cohort (i.e. beyond one year) include those not experiencing the specific event during the first year.

A per-protocol assessment of the primary endpoint was performed as sensitivity analysis. The results showed a consistent lack of association between beta-blockers and the primary outcome both within the first year and beyond the first year period (see Supplementary material online, *Table S2*). The incidence of primary outcome within and beyond the first year in the different pre-specified subgroups is shown in Supplementary material online, *Figures S1* and *S2*. Although hypothesis-generating, there was treatment heterogeneity based on diabetes status within the first year (see Supplementary material online, *Figure S1*), as well as according to the type of MI after the first year (see Supplementary material online, *Figure S2*).

### Landmark analysis of primary endpoint by LVEF

**Figure 3:**
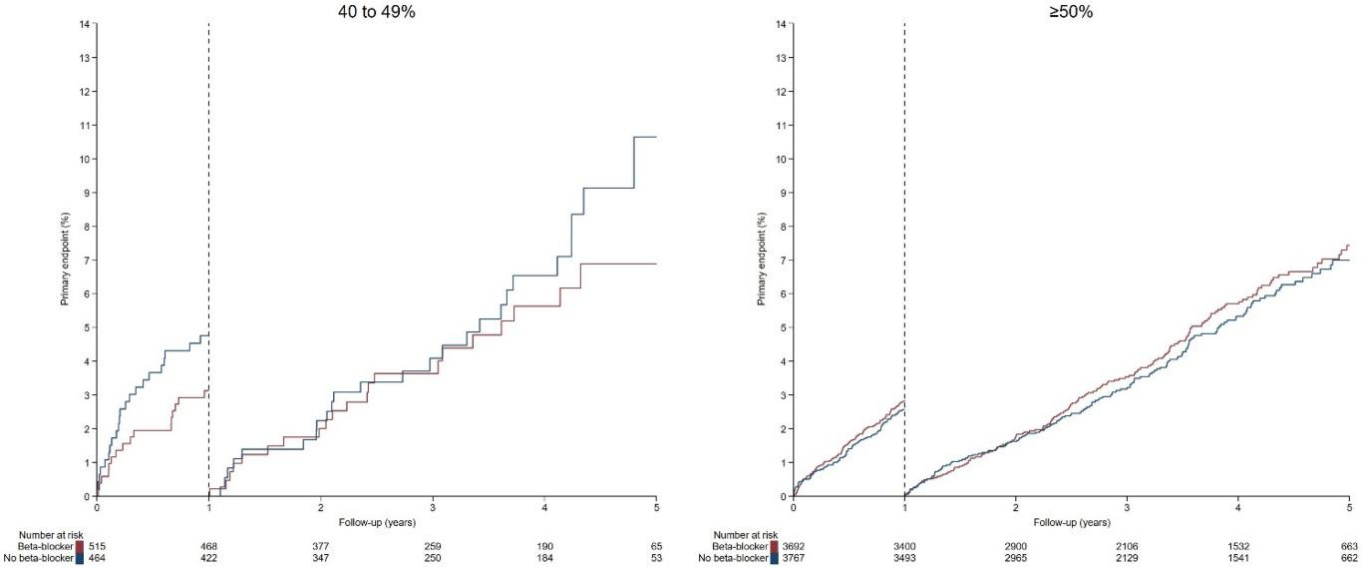
Kaplan–Meier plot for primary endpoint landmark analysis at 1 year by LVEF.

In patients with preserved LVEF (>50%), the primary endpoint occurred in 104 participants (28.9/1000 patient-years) in the beta-blocker group and in 96 patients (26.0/1000 patient-years) in the no beta-blocker group (HR 1.11; 95% CI, 0.84 to 1.46) within the first year. Beyond the first year post-MI, the primary composite endpoint happened in 173 participants (19.6/1000 patient-years) in the beta-blocker group and in 163 patients (18.1/1000 patient-years) in the no beta-blocker group (HR 1.08; 95% CI, 0.87 to 1.34). Survival curves according to the landmark are shown in *Figure 3* (left panel). Further details can be found in Supplementary material online, *Table S3*.

In patients with mildly reduced LVEF (41–49%), the primary endpoint occurred in 16 participants (32.0/1000 patient-years) in the beta-blocker group and in 22 patients (49.3/1000 patient-years) in the no beta-blocker group (HR 0.65; 95% CI, 0.34 to 1.24). Beyond the first year post-MI, the primary composite endpoint happened in 23 participants (20.9/1000 patient-years) in the beta-blocker group and in 26 patients (25.3/1000 patient-years) in the no beta-blocker group (HR 0.82; 95% CI, 0.47 to 1.43). Survival curves according to the landmark are shown in *Figure 3* (right panel).

There was no evidence that the effect of beta-blockers differed between patients with preserved vs. mildly reduced LVEF during the first year of follow-up (*P* for interaction = 0.13) or after the first year (*P* for interaction = 0.38).

### Landmark analysis of primary endpoint by beta-blocker dose

**Figure 4:**
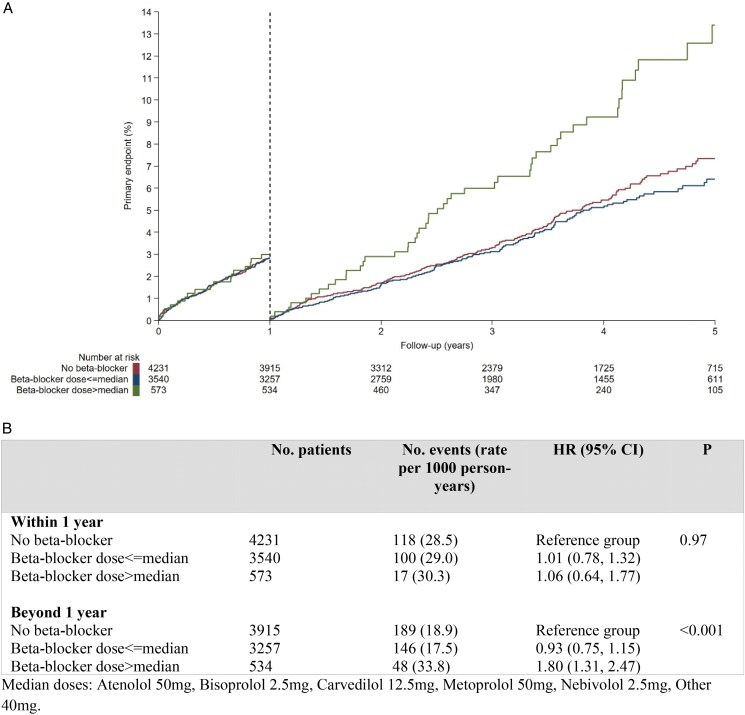
Landmark analysis at 1 year by beta-blocker dose.

Within the first year, there were no differences in risk for the primary endpoint among those not taking beta-blocker, taking a beta-blocker below or equal to the median dose, or above the median dose (*Figure 4*). In contrast, beyond year 1, those patients taking a beta-blocker dose above the median were associated with higher risk of having a primary event relative to those not taking beta-blockers (HR 1.80; 95% CI, 1.31 to 2.47; *P* < 0.001).

## Discussion

This landmark analysis of the REBOOT trial tested the potential benefits of beta-blockers after MI without reduced ejection fraction within or beyond one year after the index event. Main results of the study are:

1. Beta-blocker therapy was not associated with a reduction in clinical outcomes either within the first year after MI (ACS period) or beyond one year in patients who were event-free at that landmark (CCS period). This finding was consistent for the primary composite outcome of all-cause death, nonfatal reinfarction, or HF hospitalization, as well as for each individual outcomes including all-cause death, cardiac death, non-cardiac death, nonfatal reinfarction, HF hospitalization, stroke hospitalization, and unplanned revascularization.
2. Despite the overall prescription of secondary prevention interventions (DAPT, statins, and cardiac rehabilitation) was relatively high, patients who experienced adverse events within the first year after MI were less likely to receive these interventions compared to those with events occurring beyond the first year. Patients who remained event-free throughout the entire follow-up period had the highest rates of secondary prevention therapy prescriptions.

In this study, beta-blockers were not associated with any clinical benefit neither for ACS nor CCS patients without reduced ejection fraction.

A wide variety of mechanisms have been suggested to underlie the improved survival associated with beta-blocker therapy after MI. These include anti-ischaemic effects, prevention of reinfarction, impeding maladaptive remodelling, and reduction in arrhythmic sudden death.^1^ However, most of these benefits apply only to patients with systolic dysfunction, while the vast majority of post-MI patients do not have reduced LVEF or HF.^17^ Yet, international cohorts of real-world patients show that most post-MI patients ejection fraction are on beta-blockers regardless of the presence of these clinical features.^12,13,17–19^ While many consider beta-blockers as safe and inexpensive, they do have well-known side effects, including hypotension, bradycardia, and sexual dysfunction.^1^ Moreover, adherence to other evidence-based secondary preventive medications may decline as a result of concomitant use of beta-blockers.^20^ A revision of the effects on beta-blockers in the ACS and CCS settings was an unmet clinical need because of the lack of contemporary RCTs supporting these recommendations.

The REDUCE-AMI^6^ and REBOOT^5^ trials, encompassing a combined total of over 13 500 patients, have demonstrated that beta-blocker therapy prescribed at discharge following an uncomplicated MI without reduced ejection fraction does not confer clinical benefit. These findings provide solid evidence that beta-blocker therapy offers no clinical benefit when initiated at discharge in patients with acute coronary syndrome and preserved ejection fraction. However, whether beta-blockers provide benefit in the CCS context—i.e. beyond one year post-MI—remained a subject of debate. This question gained renewed interest following the recent ABYSS trial, which specifically enrolled CCS patients with a median time from MI of 2.9 years (range: 1.2–6.4 years), and randomized them to continue or discontinue beta-blocker therapy.^10^ The trial did not meet its non-inferiority hypothesis for beta-blocker discontinuation. The primary outcome—a composite of all-cause death, nonfatal myocardial infarction, nonfatal stroke, or hospitalization for a cardiovascular cause—occurred more frequently in the discontinuation group than in those who continued beta-blockers (HR: 1.16; 95% CI: 1.01–1.33). However, it is important to note that this difference was primarily driven by the ‘soft’ endpoint of hospitalization for cardiovascular cause. The key secondary outcome—composite of death, reinfarction, stroke, or HF hospitalization—did not differ between the groups (HR 1.11; 0.88–1.39).

Thus, the present study is consistent with ABYSS in showing that beta-blocker therapy in the CCS setting does not offer a meaningful clinical benefit in terms of reducing hard cardiovascular events.

Another relevant finding from the present study is that in the CCS context, patients taking higher doses of beta-blockers (i.e. above the median) have significantly higher incidence of the primary outcome than patients not taking beta-blockers or taking a dose below the median. There has not been a single RCT of beta-blockers in ACS and CCS patients testing for multiple doses.^21^ A few observational attempts have tried to address this issue. In the OBTAIN (Outcomes of Beta-Blocker Therapy After Myocardial Infarction) registry, 3004 post-MI one-year survivors with beta-blocker dose status available were assessed.^21^ The study found that higher doses closer to those used in pivotal RCTs did not confer greater benefit compared with lower doses. Notably, this cohort included patients regardless of LVEF, with a mean LVEF below 50%. Using the CRUSADE (Can Rapid Risk Stratification of Unstable Angina Patients Suppress Adverse Outcomes With Early Implementation of the ACC/AHA Guidelines) registry,^22^ a landmark analysis was performed in older post-MI patients who were alive and without recurrent MI after 3 years of the index MI. The cohort was stratified by no beta-blocker, <50% of target dose (according to pivotal old RCTs), and ≥50% of target dose to assess its impact on the cardiovascular composite of all-cause mortality, recurrent MI, ischaemic stroke, or HF over the subsequent 5 years. Of the 4980 patients on beta-blockers, 43% of these were treated with ≥50% of the target beta-blocker dose. They found a statistical trend towards a higher number of events in the ≥50% dose relative to the <50 dose group (54.2% vs. 50.8%, *P* = 0.10).^22^ In a similar line, we found a poorer outcome among those CCS patients on the higher beta-blocker dose relative to dose not on beta-blocker. Our findings might be different because we only included patients with a LVEF >40%, unlike the two mentioned studies, and because our subgroup classification was not based on the target dose but on the median dose of the study population. In any case, stratified analyses from two recent individual patient data meta-analyses in both mildly reduced and preserved LVEF post-MI patients seems to reinforce the idea that higher doses of beta-blockers are associated with poorer outcomes regardless of treatment efficacy.^23,24^

We evaluated the effect of beta-blocker therapy during the first year and beyond according to categories of LVEF [mildly reduced (41–49%) and preserved (≥50%)]. Our findings indicate that the subgroup at highest risk of experiencing a primary outcome event during the first year after MI consisted of patients with mildly reduced LVEF. In this group, beta-blocker therapy was associated with a numerically lower incidence of events compared with no beta-blocker therapy, although this difference did not reach statistical significance, likely due to the size of the subgroups. Nevertheless, these results appear biologically plausible. Importantly, this subgroup remains understudied, as no randomized clinical trials with adequate power have specifically addressed the efficacy of beta-blockers in the mildly reduced LVEF population—a category formally defined only in 2014.^25^ In the absence of trial-based evidence, a contemporary meta-analysis of predominantly observational studies suggested a significant reduction in mortality with beta-blocker therapy in patients with mildly reduced LVEF, while also reporting an increased risk of major cardiovascular events in those with preserved LVEF receiving beta-blockers.^12^

In the present study, we found that patients who experienced a primary outcome event during the first year after MI were, overall, less frequently prescribed secondary prevention therapies, including DAPT, statins, and cardiac rehabilitation. Interestingly, patients who developed an event beyond the first year had higher rates of these interventions compared to those with early events, though still lower than event-free patients. It is important to highlight that the prescription of these therapies at discharge after an acute coronary syndrome is a widely recognized quality-of-care indicator.^26^

Our study has relevant clinical implications and helps complete the current understanding of which patient populations with ischaemic heart disease benefit from beta-blocker therapy. While strong evidence supports the use of beta-blockers in patients with LV systolic dysfunction to improve clinical outcomes, and in those with angina to relieve symptoms, there has been a knowledge gap regarding their role in post-MI patients without these features. The present study, together with recent evidence from the REDUCE-AMI, REBOOT, and ABYSS trials,^6,5,10^ suggests that beta-blockers do not provide a benefit in terms of hard clinical events in either ACS or CCS populations without reduced ejection fraction or angina. These findings indicate that current recommendations for beta-blocker use in these patient groups may need to be reconsidered in future clinical practice guidelines.

Another relevant implication is that patients who are still taking beta-blockers long after MI (CCS) might be considered for deprescription and simplification of pharmacotherapy, unless they receive the treatment for other reasons. In the ABYSS trial, interruption of β-blocker treatment after an uncomplicated MI led to a sustained increase in blood pressure, especially in patients with history of hypertension.{Procopi, 2025 #15919}

### Limitations

The strongest point of our study are the large number of patients and events by period, as well as the uniqueness of having contemporary patients with uncomplicated MI without reduced LVEF. However, our study also has some limitations. First, although we preserved the randomization factor in the analysis and use prespecified endpoints, this landmark analysis is a *post hoc* study. Incomplete adherence and dose heterogeneity may have affected treatment effects. Second, our primary findings were based on the original randomization (e.g. whether patients were on beta-blockers at baseline) disregarding whether patients remained on the same treatment over follow-up. However, our per-protocol assessment evaluating those adhering to the original treatment allocation did not yield different findings. Treatment effect in Cohort 2 is conditional on being event-free at 1 year and should be interpreted as such. Third, some subgroup analysis had a small number of events and might be underpowered to make reliable conclusions. A sample size estimation was not performed before assessing the data. Moreover, subgroups were defined using baseline values because these variables were not collected at follow-up visits. Finally, no adjustment for multiplicity was performed. However, this might have little impact on our main conclusions given that the alternative hypothesis has been largely rejected in most of our comparisons.^16^

## Conclusions

In this landmark analysis of the REBOOT trial, which recruited patients with uncomplicated MI invasively managed without reduced LVEF, beta-blocker use was not associated with an improvement in clinical outcomes in neither its ACS (<1 year) nor its derived CCS population (>1-year follow-up in those event-free at this landmark). This was consistent both for the primary composite outcome of all-cause death, non-fatal reinfarction or HF admission, as well as for each individual outcomes of all-cause death, cardiac death, non-cardiac death, nonfatal reinfarction, HF admission, admission for stroke, and unplanned revascularization. Moreover, among the CCS population, those with a beta-blocker dose above the median had a worse prognosis than those not taking beta-blockers. Overall, despite relatively high rates of secondary prevention interventions prescriptions, patients who experienced an adverse event within the first year after MI were less frequently prescribed these therapies compared to those who had events beyond one year, and both groups had lower prescription rates than patients who remained event-free throughout follow-up.

## Supplementary Material

pvag002_Supplementary_Data

## Data Availability

The data underlying this article will be shared on reasonable request to the corresponding author.
