# Supplementary material for: Effect of beta blockers in acute and chronic coronary syndromes without reduced ejection fraction: a landmark analysis from the REBOOT trial
Source: Eur Heart J Cardiovasc Pharmacother. 2026 Jan 22;12(2):64–74. doi: 10.1093/ehjcvp/pvag002 (PMC12946967; doi:10.1093/ehjcvp/pvag002)

Supplementary material

Table S1. Landmark analysis at 1 year of other endpoints

		N (rate per 1000 person-years)		Rate difference (95% CI)	HR (95% CI)	P
		Beta-blocker	No beta-blocker			
**Other endpoints**						
Sustained ventricular tachycardia	Within 1 year	2 (0.5)	0 (0.0)	0.48 (-0.19, 1.15)	7.2e+14 (0.00, .)	0.16
	Beyond 1 year	1 (0.1)	2 (0.2)	-0.10 (-0.43, 0.23)	0.51 (0.05, 5.59)	0.57
Ventricular fibrillation	Within 1 year	1 (0.2)	2 (0.5)	-0.24 (-1.05, 0.58)	0.50 (0.05, 5.56)	0.57
	Beyond 1 year	2 (0.2)	3 (0.3)	-0.09 (-0.52, 0.33)	0.67 (0.11, 4.03)	0.66
Resuscitated cardiac arrest	Within 1 year	1 (0.2)	0 (0.0)	0.24 (-0.23, 0.71)	7.2e+14 (0.00, .)	0.32
	Beyond 1 year	3 (0.3)	4 (0.4)	-0.09 (-0.60, 0.41)	0.76 (0.17, 3.39)	0.72
Admission for symptomatic advanced AV block	Within 1 year	3 (0.7)	2 (0.5)	0.25 (-0.81, 1.30)	1.51 (0.25, 9.06)	0.65
	Beyond 1 year	4 (0.4)	4 (0.4)	0.00 (-0.54, 0.54)	1.01 (0.25, 4.03)	0.99

Table S2. Landmark per-protocol analysis at 1 year of the primary endpoint where patients are censored at the visit a crossover is reported

		N (rate per 1000 person-years)		Rate difference (95% CI)	HR (95% CI)	P
		**Beta-blocker**	**No beta-blocker**			
**Primary endpoint**						
All-cause death, nonfatal reinfarction and heart failure admission	Within 1 year	102 (25.6)	85 (21.9)	3.75 (-3.05, 10.56)	1.17 (0.88, 1.57)	0.27
	Beyond 1 year	176 (20.4)	175 (21.1)	-0.72 (-5.06, 3.62)	0.97 (0.79, 1.19)	0.76

Hazard ratios were estimated using Cox proportional hazards models and estimate the effect of taking beta-blocker vs no beta-blocker. P values were calculated using logrank tests.

Table S3. Landmark analysis at 1 year of the primary endpoint by LVEF

		N (rate per 1000 person-years)		Rate difference (95% CI)	HR (95% CI)	P
		Beta-blocker	No beta-blocker			
**LVEF**						
>=50%	Within 1 year	104 (28.9)	96 (26.0)	2.85 (-4.77, 10.46)	1.11 (0.84, 1.46)	0.46
	Beyond 1 year	173 (19.6)	163 (18.1)	1.49 (-2.55, 5.52)	1.08 (0.87, 1.34)	0.47
41-49%	Within 1 year	16 (32.0)	22 (49.3)	-17.36 (-43.26, 8.53)	0.65 (0.34, 1.24)	0.19
	Beyond 1 year	23 (20.9)	26 (25.3)	-4.41 (-17.37, 8.55)	0.82 (0.47, 1.43)	0.48

Figure S1. Subgroup analysis of the primary composite outcome all-cause death, nonfatal reinfarction and heart failure admission within 1 year after myocardial infarction (acute coronary syndrome period)

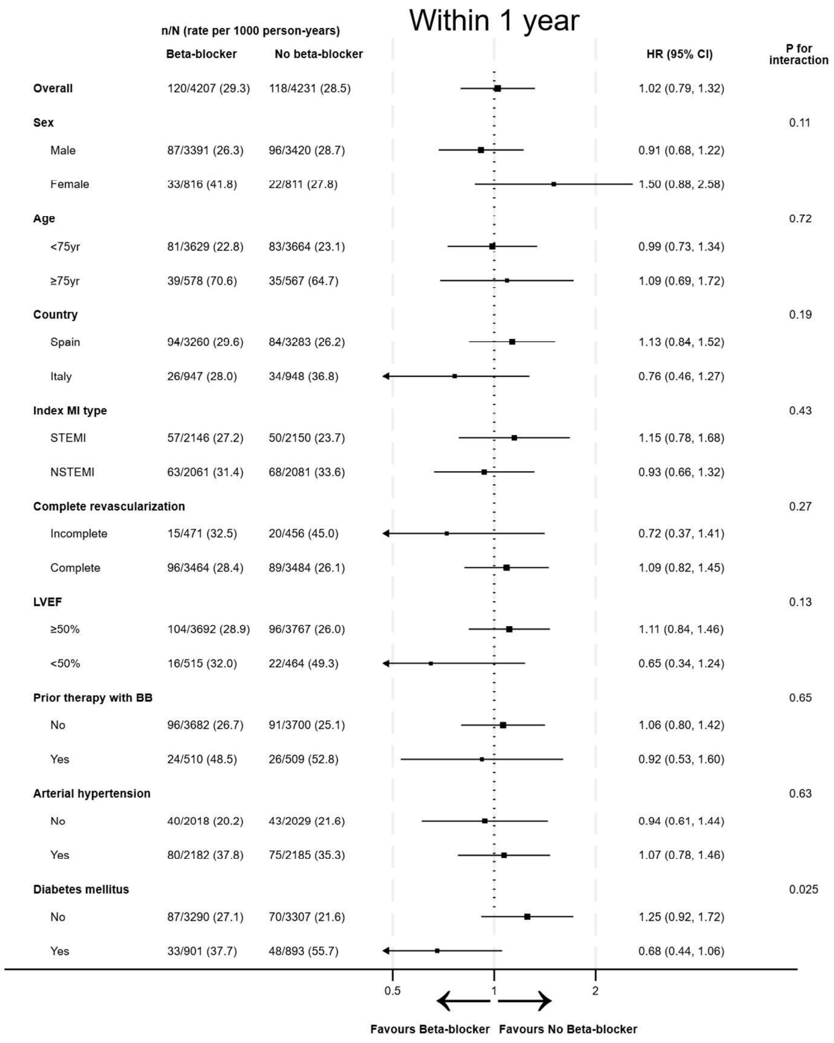

Figure S2. Subgroup analysis of the primary composite outcome all-cause death, nonfatal reinfarction and heart failure admission after 1 year after myocardial infarction (chronic coronary syndrome period)

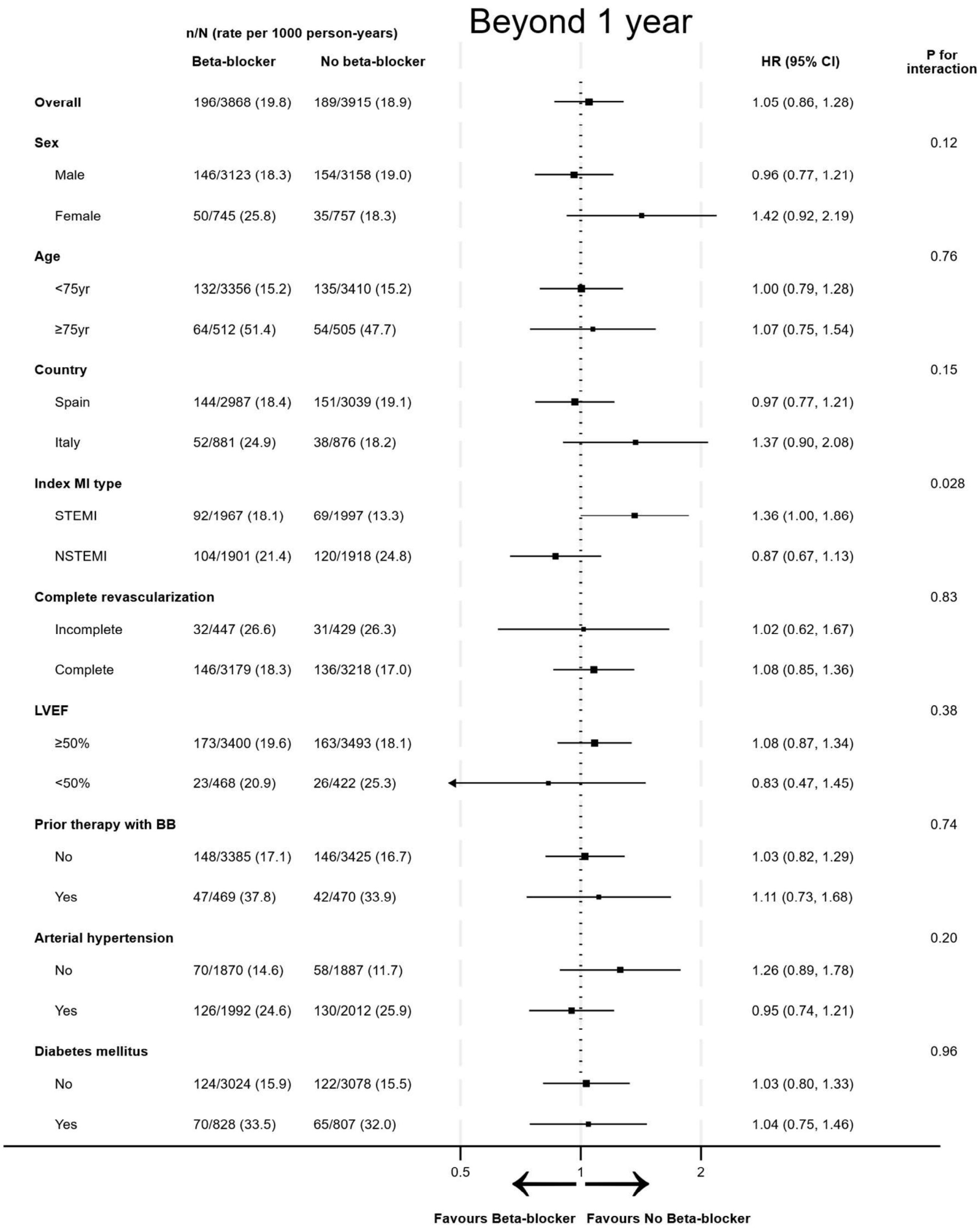

Supplement: pvag002_Supplementary_Data [file pvag002_supplementary_data.pdf]
